# Supplementary material for: Projections from thalamic nucleus reuniens to hippocampal CA1 area participate in context fear extinction by affecting extinction-induced molecular remodeling of excitatory synapses
Source: eLife. 2025 Jan 23;13:RP101736. doi: 10.7554/eLife.101736 (PMC11756855; doi:10.7554/eLife.101736)
Supplement: MDAR checklist [file elife-101736-mdarchecklist1.pdf]

eLife

Materials Design Analysis Reporting (MDAR) Checklist for Authors

The MDAR framework establishes a minimum set of requirements in transparent reporting mainly applicable to studies in the life sciences.

eLife asks authors to **provide detailed information within their article** to facilitate the interpretation and replication of their work. Authors can also upload supporting materials to comply with relevant reporting guidelines for health-related research (see EQUATOR Network), life science research (see the BioSharing Information Resource), or animal research (see the ARRIVE Guidelines and the STRANGE Framework; for details, see *eLife*'s Journal Policies). Where applicable, authors should refer to any relevant reporting standards materials in this form.

For all that apply, please note **where in the article** the information is provided. Please note that we also collect information about data availability and ethics in the submission form.

Materials:

**Newly created materials**	**Indicate where provided: section/figure legend**	**N/A**
The manuscript includes a dedicated "materials availability statement" providing transparent disclosure about availability of newly created materials including details on how materials can be accessed and describing any restrictions on access.	N/A	

**Antibodies**	**Indicate where provided: section/figure legend**	**N/A**
For commercial reagents, provide supplier name, catalogue number and RRID, if available.<br><br>Anti-Post Synaptic Density Protein 95, clone 7E3-1B8 (Millipore Cat# MAB1598, RRID:AB_94278)<br>Donkey anti-Mouse IgG (H+L) Highly Cross-Adsorbed Secondary Antibody, Alexa Fluor™ 647 (Thermo Fisher Scientific Cat# A-31571, RRID:AB_162542)	Materials and Methods. : Immunostaining.	

**DNA and RNA sequences**	**Indicate where provided: section/figure legend**	**N/A**
Short novel DNA or RNA including primers, probes: Sequences should be included or deposited in a public repository.	N/A	

Cell materials	Indicate where provided: section/figure legend	N/A
Cell lines: Provide species information, strain. Provide accession number in repository OR supplier name, catalog number, clone number, OR RRID.	N/A	
Primary cultures: Provide species, strain, sex of origin, genetic modification status.	N/A	

Experimental animals	Indicate where provided: section/figure legend	N/A
Laboratory animals or Model organisms: Provide species, strain, sex, age, genetic modification status. Provide accession number in repository OR supplier name, catalog number, clone number, OR RRID.<br><br>We used 3-4 month old male and female mice of two strains:<br>**Thy1-GFP(M)** (The Jackson Laboratory, RRID:IMSR_JAX:007788)<br>**C57BL/6J** (The Jackson Laboratory, RRID:IMSR_JAX:000664)/ Białystok University, Poland	Materials and Methods. Animals.	
Animal observed in or captured from the field: Provide species, sex, and age where possible.		

Plants and microbes	Indicate where provided: section/figure legend	N/A
Plants: provide species and strain, ecotype and cultivar where relevant, unique accession number if available, and source (including location for collected wild specimens).	N/A	
Microbes: provide species and strain, unique accession number if available, and source.	N/A	

Human research participants	Indicate where provided: section/figure legend) or state if these demographics were not collected	N/A
If collected and within the bounds of privacy constraints report on age, sex, gender and ethnicity for all study participants.	N/A	

Design:

Study protocol	Indicate where provided: section/figure legend	N/A

If the study protocol has been pre-registered, provide DOI. For clinical trials, provide the trial registration number OR cite DOI.	N/A	

**Laboratory protocol**	**Indicate where provided: section/figure legend**	**N/A**
Provide DOI OR other citation details if detailed step-by-step protocols are available.	N/A	

**Experimental study design (statistics details) ***		
**For in vivo studies: State whether and how the following have been done**	**Indicate where provided: section/figure legend. If it could have been done, but was not, write "not done"**	**N/A**
Sample size determination<br>As the size effect of fear extinction on synapse remodelling in the RE→CA1 pathway was unknown, sample size in our experiments was determined based on published experiments and our experience in conducting similar studies.	Materials and methods. *Statistics*	
Randomisation<br>We used both males and females in age- and sex-balanced groups.	Materials and methods. *Animals*.	
Blinding<br>The experimenters were not blind to the experimental groups during in vivo behavioural studies but mice behaviour was scored automatically by the software. In all other cases the animals/samples were coded.	Materials and methods.<br>*Confocal microscopy and image quantification.*<br>*Serial Block-FaceScanning Electron Microscopy (SBFSEM)*.	
Inclusion/exclusion criteria<br>The samples/animals were excluded from the analysis only when evident technical problems occurred during experiments.	Materials and methods. *Statistics*	

**Sample definition and in-laboratory replication**	**Indicate where provided: section/figure legend**	**N/A**
State number of times the experiment was replicated in the laboratory.<br><br>All experiments were conducted once, except for the experiments shown on Figure 6. This is described in the Results section	Results. Figure 6.	
Define whether data describe technical or biological replicates.<br><br>Each figure legend contains a description of presented data.	Figure 1-7 legends.	

In most cases we used N=biological replicate (animal).		

**Ethics**	**Indicate where provided: section/submission form**	**N/A**
Studies involving human participants: State details of authority granting ethics approval (IRB or equivalent committee(s), provide reference number for approval.	N/A	
Studies involving experimental animals: State details of authority granting ethics approval (IRB or equivalent committee(s), provide reference number for approval.<br><br>All experiments were undertaken in accordance with the Poland Animals (Scientific Procedures) Act and approved by the Local Ethics Committee in Warsaw, Poland (no. 529/2018).	Materials and methods. *Animals*.	
Studies involving specimen and field samples: State if relevant permits obtained, provide details of authority approving study; if none were required, explain why.	N/A	

**Dual Use Research of Concern (DURC)**	**Indicate where provided: section/submission form**	**N/A**
If study is subject to dual use research of concern regulations, state the authority granting approval and reference number for the regulatory approval.	N/A	

Analysis:

**Attrition**	**Indicate where provided: section/figure legend**	**N/A**
Describe whether exclusion criteria were pre-established. Report if sample or data points were omitted from analysis. If yes, report if this was due to attrition or intentional exclusion and provide justification.<br><br>The samples/animals were excluded from the analysis only when evident technical problems occurred while processing the samples or training the animals.	Materials and methods. *Statistics*	

**Statistics**	**Indicate where provided: section/figure legend**	**N/A**
Describe statistical tests used and justify choice of tests.<br><br>Data with normal distribution are presented as mean ± standard error of the mean (SEM) or as median ±	Materials and methods. *Statistics*	

interquartile range (IQR) for the population with non-normal distribution. When the data met the assumptions of parametric statistical tests, results were analyzed by one- or repeated measures two-way ANOVA, followed by Tukey's or Fisher's *post hoc* tests, where applicable. Areas of axonal boutons, dendritic spines and PSD-95 puncta did not follow normal distributions and were analyzed with the Kruskal-Wallis test. To facilitate the interpretation of our results, we followed the convention of defining $p < 0.05$ as significant in the text. Wherever possible, we used estimation-based statistics with mean-difference plots instead [37].		

**Data availability**	**Indicate where provided: section/submission form**	**N/A**
For newly created and reused datasets, the manuscript includes a data availability statement that provides details for access (or notes restrictions on access).	N/A	
When newly created datasets are publicly available, provide accession number in repository OR DOI and licensing details where available.<br><br>Row data and the code used for analysis of confocal data is available at OSF (https://osf.io/bnkpx/).	**DATA AVAILABILITY**	
If reused data is publicly available provide accession number in repository OR DOI, OR URL, OR citation.	N/A	

**Code availability**	**Indicate where provided: section/figure legend**	**N/A**
For any computer code/software/mathematical algorithms essential for replicating the main findings of the study, whether newly generated or re-used, the manuscript includes a data availability statement that provides details for access or notes restrictions.	N/A	
Where newly generated code is publicly available, provide accession number in repository, OR DOI OR URL and licensing details where available. State any restrictions on code availability or accessibility.<br><br>Row data and the code used for analysis of confocal data is available at OSF (https://osf.io/bnkpx/).	**DATA AVAILABILITY**	
If reused code is publicly available provide accession number in repository OR DOI OR URL, OR citation.	N/A	

Reporting:

The MDAR framework recommends adoption of discipline-specific guidelines, established and endorsed through community initiatives.

Adherence to community standards	Indicate where provided: section/figure legend	N/A
State if relevant guidelines (e.g., ICMJE, MIBBI, ARRIVE, STRANGE) have been followed, and whether a checklist (e.g., CONSORT, PRISMA, ARRIVE) is provided with the manuscript.<br><br>**ARRIVE**	N/A	

---

* We provide the following guidance regarding transparent reporting and statistics; we also refer authors to Ten common statistical mistakes to watch out for when writing or reviewing a manuscript.

Sample-size estimation

- You should state whether an appropriate sample size was computed when the study was being designed
- You should state the statistical method of sample size computation and any required assumptions
- If no explicit power analysis was used, you should describe how you decided what sample (replicate) size (number) to use

Replicates

- You should report how often each experiment was performed
- You should include a definition of biological versus technical replication
- The data obtained should be provided and sufficient information should be provided to indicate the number of independent biological and/or technical replicates
- If you encountered any outliers, you should describe how these were handled
- Criteria for exclusion/inclusion of data should be clearly stated
- High-throughput sequence data should be uploaded before submission, with a private link for reviewers provided (these are available from both GEO and ArrayExpress)

Statistical reporting

- Statistical analysis methods should be described and justified
- Raw data should be presented in figures whenever informative to do so (typically when N per group is less than 10)
- For each experiment, you should identify the statistical tests used, exact values of N, definitions of center, methods of multiple test correction, and dispersion and precision measures (e.g., mean, median, SD, SEM, confidence intervals; and, for the major substantive results, a measure of effect size (e.g., Pearson's r, Cohen's d)
- Report exact p-values wherever possible alongside the summary statistics and 95% confidence intervals. These should be reported for all key questions and not only when the p-value is less than 0.05.

Group allocation

- Indicate how samples were allocated into experimental groups (in the case of clinical studies, please specify allocation to treatment method); if randomization was used, please also state if restricted randomization was applied
- Indicate if masking was used during group allocation, data collection and/or data analysis
